# Multi-scale feature progressive fusion network for remote sensing image change detection

**DOI:** 10.1038/s41598-022-16329-6

**Published:** 2022-07-13

**Authors:** Di Lu, Shuli Cheng, Liejun Wang, Shiji Song

**Affiliations:** 1 College of Information Science and Engineering, Xinjiang University, Urumqi, 830046 China; 2 Department of Automation, Tsinghua University, Beijing, 100084 China

**Keywords:** Imaging, Electrical and electronic engineering

## Abstract

Presently, research on deep learning-based change detection (CD) methods has become a hot topic. In particular, feature pyramid networks (FPNs) are widely used in CD tasks to gradually fuse semantic features. However, existing FPN-based CD methods do not correctly detect the complete change region and cannot accurately locate the boundaries of the change region. To solve these problems, a new Multi-Scale Feature Progressive Fusion Network (MFPF-Net) is proposed, which consists of three innovative modules: Layer Feature Fusion Module (LFFM), Multi-Scale Feature Aggregation Module (MSFA), and Multi-Scale Feature Distribution Module (MSFD). Specifically, we first concatenate the features of each layer extracted from the bi-temporal images with their difference maps, and the resulting change maps fuse richer semantic information while effectively representing change regions. Then, the obtained change maps of each layer are directly aggregated, which improves the effective communication and full fusion of feature maps in CD while avoiding the interference of indirect information. Finally, the aggregated feature maps are layered again by pooling and convolution operations, and then a feature fusion strategy with a pyramid structure is used, with layers fused from low to high, to obtain richer contextual information, so that each layer of the layered feature maps has original semantic information and semantic features of other layers. We conducted comprehensive experiments on three publicly available benchmark datasets, CDD, LEVIR-CD, and WHU-CD to verify the effectiveness of the method, and the experimental results show that the method in this paper outperforms other comparative methods.

## Introduction

Remote sensing image change detection (CD) is essentially the detection of surface change information at different time stages, and this technology has important applications and research values in real life. In recent years, with the rapid development of technology, especially the implementation of high-resolution surface observation projects, satellite remote sensing technology has entered the era of sub-meter level. Remote sensing technology is flourishing in various fields, among which remote sensing image CD has received wide attention from many scholars at home and abroad because of its important role in environmental monitoring, disaster assessment, urban research and so on. Most of the traditional remote sensing image CD methods^1–5^ require a priori knowledge of the domain, and these CD methods are designed and extracted manually by features such as texture, morphology, and neighborhood information of the images for change region inference, and they extract shallow image features, which are difficult to capture high-level semantic information and time-consuming. Recently, convolutional neural networks (CNNs) have greatly advanced the development of CD methods^6–12^ due to their ability to extract both low-level details and high-level semantic information. Many remote sensing image CD methods based on deep learning^13–22^ have achieved great success, and their detection results are better than those of traditional methods^23–28^. In their experiments, scientists found that the methods of using feature pyramid networks (FPNs^29,30^) with fusion of high and low-level features are significantly effective in fully extracting object features and improving the accuracy of boundary details. Therefore, many advanced CD methods^15,31,32^ are composed of feature fusion structures similar to FPNs.

Although FPN-like feature fusion models have achieved remarkable results in the field of computer vision, they still have some shortcomings. On the one hand, as mentioned in the paper^33^, in the pyramid feature fusion structure, the deep feature information is transferred to the shallow features layer by layer. Therefore, the proportion of feature information carried by deep features in the entire fusion continues to decrease. On the other hand, in the final stage, low-level features containing rich spatial information are introduced into the network, obtaining predicted change maps with boundary information. Finally, the predicted change maps may have ambiguous object boundary information or the predicted foreground information with too much weight. To solve the above problems, Zhang et al.^32^ designed a dynamic fusion model. The model adaptively learns target feature information from the input of the model by introducing a dynamic convolution module and utilizes multi-layer supervision to train the network. In addition, the model focuses on learning both high-level and low-level feature information. Zhang et al.^34^ provided a coarse-to-fine CD model, which can effectively solve the problem of excessive weight of foreground information prediction through two-stage feature fusion. Some CD models^10,17,34^ adopt the attention mechanism to improve network performance. These models first extract the rich information in the feature map and then gradually integrate the contextual feature information. Although these models ensure rich information fusion, these models still have problems similar to FPN-like.

In this article, a novel Multi-Scale Feature Progressive Fusion Network (MFPF-Net) is proposed for remote sensing image CD, which aims to fully fuse bi-temporal remote sensing images, exchange feature information, promote information propagation and achieve better detection results. In MFPF-Net, the features extracted from the backbone network are first fed into the Layer Feature Fusion Module (LFFM), which integrates the pre- and post-change feature information, thus improving the network's ability to identify the changed regions. The MFPF-Net also contains a Multi-Scale Feature Aggregation Module (MSFA), which adaptively assigns weights to the information in the features at different stages and allows communication between the different stages. Finally, a Multi-Scale Feature Distribution Module (MSFD) is designed to fully extract the semantic and detailed information of the features through a distributed approach. In summary, the MFPF-Net network can clearly and completely detect the detailed information of the change region and achieve good results. Our main contributions are summarized as follows:

1. To maximize the difference feature information of the change maps and detect the complete change regions and edge information, a novel layer feature fusion module (LFFM) is proposed, which consists of two consecutive residual blocks. the LFFM concatenates the layer bi-temporal feature pairs output by the feature extraction network with their difference maps so that the semantic information of the bi-temporal feature pairs can be fully fused. A higher-quality change map is obtained while retaining rich semantic information.
2. We design the Multi-Scale Feature Aggregation Module (MSFA), which directly aggregates the change features of different layers obtained by the LFFM and adaptively predicts a set of weights according to the different importance of the features of each layer, which avoids the loss of some information brought by indirect fusion and enables the semantic information between different stages to communicate and fuse directly, and according to the effectiveness of different stage. The importance of different stage features is weighed according to their effectiveness in identifying regions of change.
3. To obtain more satisfactory prediction results, a Multi-Scale Feature Distribution (MSFD) module is proposed. The MSFD module allocates multi-level features by using multi-scale pooling and convolution operations on the feature maps output from the MSFA module, combines these feature maps with the progressive structure of FPN, and detects change regions in a stage-wise fusion. In this way, both semantic and change region details can be accessed adaptively at each level of fusion, which facilitates the stage-wise fusion and helps to better predict the change region and obtain a higher quality prediction result.

The extensive experiments conducted on CDD^16^, LEVIR-CD^17^, and WHU-CD^35^ proved that MFPF-Net outperforms the state-of-the-art (SOTA) methods. In particular, the F1 of MFPF-Net reaches 95.9% on the CDD dataset.

This paper is organized as follows. The “Related work” presents the background related to the research content of this article. In "Methodology", the proposed approach is described in detail. In "Experiments and analysis", comparative and ablation experiments on these three popular open-source datasets are shown, and the performance and efficiency of the proposed model are visualized. In "Conclusion", general conclusions are made about the work of this paper.

## Related work

The existing CD methods can be roughly classified into traditional methods and deep learning-based methods, and each will be briefly introduced in the following sections.

### Traditional methods

Traditional methods need to manually set parameters or thresholds when using remote sensing images for CD. Artificially designed features only achieve good results in specific scenes. The features designed manually by a priori knowledge are not representative and have poor generalization performance, which makes it impossible to achieve good results in CD of high-resolution remote sensing images, and it is not suitable for CD in complex scenes. Traditional CD methods can be divided into pixel-level CD methods and object-level CD methods^1^. The pixel-level methods^2,3,24,25^ are mainly suitable for remote sensing images with medium and low resolution. These methods mainly calculate the difference of the corresponding pixel values and obtain the change maps based on these differences by simply setting the threshold or clustering. Because the fusion of contextual information is ignored in the model, it may cause the model to extract deep features while ignoring shallow information. Simultaneously, with the development of a series of high-resolution optical sensors, high-resolution images contain a wealth of information. The object-level CD methods are proposed for high-resolution CD. The object-level methods^4,5,24^ divide the image into objects and then compare and analyze the objects in the bi-temporal image by extracting rich geometric information and spectral information in the images.

### Deep-learning-based methods

With the rapid development of deep learning technology in computer vision, remote sensing image CD has made great success in accuracy improvement with deep learning. In recent years, deep learning features with rich semantic information have been introduced to replace the low-level manual design features. Some methods use the deep convolution neural network (CNN) as the feature extractor, rather than using the descriptors that require a large number of domain knowledge designed by human beings. Due to its strong detection ability, CNN has successfully achieved great success in remote sensing image CD tasks. The CD methods^10–15^ use CNN structure to extract rich features from bi-temporal images and obtain the final change map, which has achieved good results. In 2015, Gong et al.^36^ proposed a synthetic aperture radar CD network, which can generate difference maps with good detection performance. Subsequently, CDNet^37^ uses an image pair as input, uses the SLAM system of multi-sensor fusion, and combined it with the density 3D reconstruction system to register the video sequence. Finally, the pixel-level structure change maps are obtained. In addition, this paper creates a new urban CD dataset. Daudt et al.^38^ proposed three fully convolutional networks (FCN)^39^ structures to solve the problem of CD, namely FC-EF, FC-Siam-Conc, and FC-Siam-Diff. FC-EF is based on UNet^30^ structure. FC-Siam-Conc performs skip connection operation on three feature maps from two encoder branches and the corresponding layer of the decoder. FC-Siam-Diff first obtains the absolute value of the difference between the feature maps of the two decoder branches and then performs skip connection with the corresponding layer of the decoder. STANet^17^ proposed a CD self-attention mechanism to model the spatial–temporal relationship. In this paper, two self-attention modules, BAM and PAM, are proposed. The attention weights of any two pixels at different times and positions are calculated by these two modules, and good results are achieved. DASNet^10^ captured many discriminative feature representations by using the dual attention mechanism, which improved the recognition accuracy of the network. In 2021, CLNet^31^ proposed a cross-layer network based on U-Net, which made innovations for insufficient context feature information fusion. HDFNet^32^ designed a dynamic fusion network considering the shortcomings of regional integrity detection and introduced a dynamic convolution model for adaptive learning. The network also achieved good performance. Zhang et al.^34^ introduced a CD method from coarse to fine, which is divided into the coarse detection stage and the fine detection stage. The detection of the two stages can obtain more abundant feature representations, and a mixed loss function is proposed to provide different levels of supervision. Although the above methods have achieved good performance, some features may introduce ambiguous context information for CD. How to obtain effective feature representation and fully integrate feature context information has become an urgent problem in CD.

## Methodology

### Research motivation

At present, there are still some problems in remote sensing image CD that need to be dealt with: (1) High-resolution remote sensing images are rich in spectral and spatial information, but these information have not been fully utilized; (2) most SOTA CD methods are implemented by FPN-like feature fusion structure, in the process of feature fusion, the spatial structure details used to reconstruct the object boundary can only be obtained in the final fusion stage, which makes the change map predicted by these methods have low-quality object boundary or miss detection of small change regions^41^.

The object of this article is to construct a novel remote sensing image CD network, MFPF-Net, to achieve better high-resolution detection performance. The MFPF-Net network can fully and effectively extract the bi-temporal feature information of high-resolution remote sensing images, and allow efficient information communication across multiple levels. It can detect the boundary information of the changing region more clearly, and effectively avoid the missing detection of small regions.

### Overview of the proposed MFPF-Net

**Figure 1:**
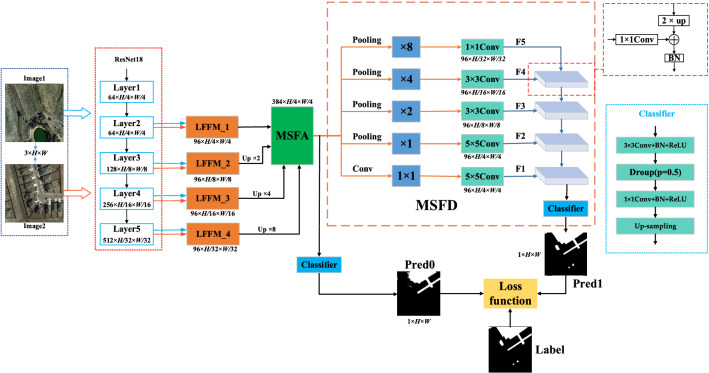
Framework of the MFPF-Net network (created by ‘Microsoft Office Visio 2013’ url: https://www.microsoft.com/zh-cn/microsoft-365/previous-versions/microsoft-visio-2013).


Figure 1 shows the overall architecture of the MFPF-Net. The whole MFPF-Net network consists of the backbone network ResNet18^40^ and three modules LFFM, MSFA and MSFD. The bi-temporal images pairs (Image1, Image2) are fed into a feature extraction network with two weight-shared ResNet18s, and the two images will output two groups of multi-scale feature maps, respectively. Then, the two feature maps with the same scale in both groups are sent together to the corresponding LFFM module for feature fusion. The fused multi-scale feature maps are fed into the MSFA module, which directly aggregates the multi-scale feature maps and then adaptively generates a set of weights to enhance the feature representation of the feature maps. The first Pred0 of the model is output after the MSFA module. After that, the aggregated feature maps updated by the MSFA are further processed by the MSFD module. MSFD uses global pooling at different scales and convolution operations with different convolution kernels to reallocate the aggregated feature maps to the corresponding layers. Finally, the layered features are fused in a top-down fusion method to obtain the second Pred1 of the network. In the training phase, the optimized network parameters are obtained by deep supervision of the model. The process of the MFPF-Net is shown in Algorithm 1. In this section, firstly, we present the overall framework of the proposed network. Then the three main novel modules are specifically discussed. Finally, we provide details of the loss function.

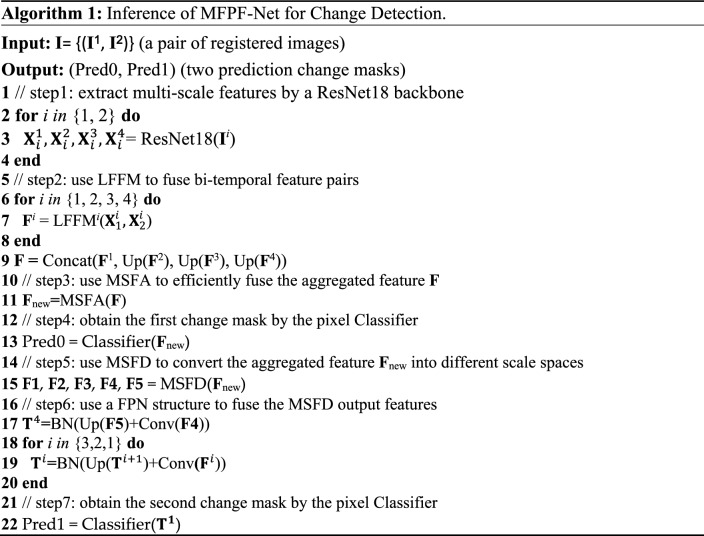


### Layer feature fusion module

At present, the feature fusion methods for CD can be divided into two types: pre- and post-fusion^42^. The pre-fusion indicates that the images obtained after concatenating the bi-temporal image pairs or their difference maps are fed into the network for feature extraction to obtain the change maps, and then CD is performed. The pre-fused images do not adequately represent the high-level semantics of the features extracted from each of the bi-temporal images fed into the network, and the pre-fused images are more sensitive to noise compared to a single original image. The post-fusion is a feature extraction process for bi-temporal images separately using the same backbone network, and then the extracted bi-temporal features are fused and change inference is performed using the CD network. However, during the actual experiments, we found that such fusion does not enable the feature maps to have both high-level semantic information and low-level semantic information.

**Figure 2:**
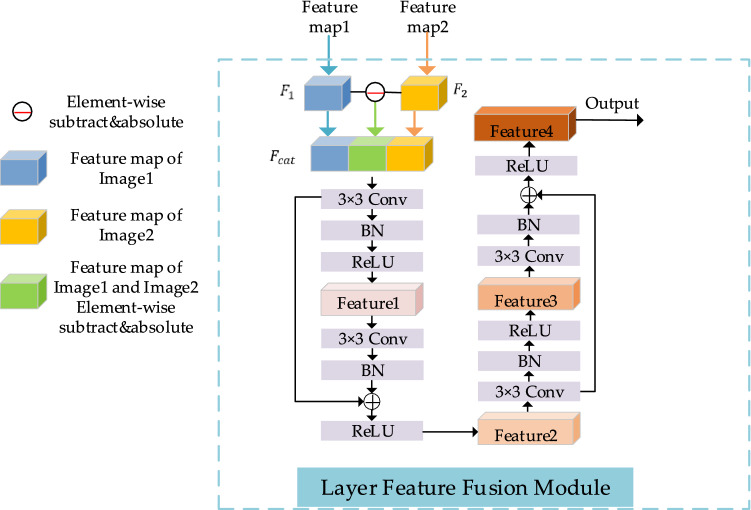
The structure of LFFM (created by ‘Microsoft Office Visio 2013’ url: https://www.microsoft.com/zh-cn/microsoft-365/previous-versions/microsoft-visio-2013).

In this paper, the advantages and disadvantages of these two methods are fully considered. We consider that each layer of the feature map output by the feature extraction network has different semantic information. To make the final change maps better represent the change regions and boundaries, we designed the LFFM, and its structure is shown in Fig. 2. We apply the LFFM to perform the concatenation operation on two feature maps ${F}_{1}$ and ${F}_{2}$ of the same layer and their difference map. Specifically, the bi-temporal image pairs (Image1 and Image2) are fed into the backbone network ResNet18 to obtain $({\mathrm{F}}_{1}^{1},{\mathrm{F}}_{1}^{2},{\mathrm{F}}_{1}^{3},{\mathrm{F}}_{1}^{4}$) and $({\mathrm{F}}_{2}^{1},{\mathrm{F}}_{2}^{2},{\mathrm{F}}_{2}^{3},{\mathrm{F}}_{2}^{4}$), respectively, and then the feature maps corresponding to the two images at each layer and their difference maps are concatenated along the channel dimension. This operation is defined as follows:

1 $${F}_{cat}=Cat\left({F}_{1},{F}_{2},Diff\left[{F}_{1},{F}_{2}\right]\right)$$

where *Cat* denotes a concatenation operation and *Diff* [.,.] denotes the difference of feature pairs and takes the absolute value. Then using two consecutive residual blocks, each residual block consists of two convolutional layers, and each convolutional layer includes $3\times 3$ Conv, BN, ReLU operations, as shown in Fig. 2. Given the input feature pairs ${\mathrm{F}}_{1}^{i}\in {\mathbb{R}}^{{C}_{i}\times {H}_{i}\times {W}_{i}}$ and ${\mathrm{F}}_{2}^{i}\in {\mathbb{R}}^{{C}_{i}\times {H}_{i}\times {W}_{i}} \left(i=\mathrm{1,2},\mathrm{3,4}\right)$, the feature ${F}_{cat}^{i}\in {\mathbb{R}}^{3{C}_{i}\times {H}_{i}\times {W}_{i}}$ is obtained after the operation shown in Eq. (1). In the first residual block, the number of channels of the feature ${F}_{cat}^{i}$ becomes ${C}_{i}$ after using the $3\times 3$ Conv operation, and then the BN and ReLU operations are completed to obtain the feature map $Feature1\in {\mathbb{R}}^{{C}_{i}\times {H}_{i}\times {W}_{i}}$. The calculation process is shown in the following equation:

2 $$Feature1=ReLU\left(BN\left(Conv\left({F}_{cat}\right)\right)\right)$$

After applying the $3\times 3$ Conv and BN operations on $Feature1$ and the feature output from the first $3\times 3$ Conv operation, the element-wise summation operation is used to obtain the feature map $Feature2\in {\mathbb{R}}^{{C}_{i}\times {H}_{i}\times {W}_{i}}$. The calculation process is shown in the following equation:

3 $$Feature2=ReLU\left(BN\left(Conv\left(Feature1\right)\right)+Conv\left({F}_{cat}\right)\right)$$

In the second residual block, the number of channels of feature $Feature2$ is changed to 96 after the $3\times 3$ Conv operation, the purpose of this process is to complete the multi-scale aggregation operation later, and also to avoid the distortion of the data caused by excessive compression of the number of channels. After the first convolution layer, the feature map $Feature3\in {\mathbb{R}}^{96\times {H}_{i}\times {W}_{i}}$ is obtained:

4 $$Feature3=ReLU\left(BN\left(Conv\left(Feature2\right)\right)\right)$$

and after the second convolution layer, the output feature $F4\in {\mathbb{R}}^{96\times {H}_{i}\times {W}_{i}}$ is obtained:

5 $$Feature4=ReLU\left(BN\left(Conv\left(Feature3\right)\right)+Conv\left(Feature2\right)\right)$$

Finally, the feature maps after the fusion of the bi-temporal features of these four stages can be obtained at Layer2, Layer3, Layer4 and Layer5.

### Multi-scale feature aggregation module

As mentioned in the previous section, FPN-based CD methods^15,25,32^ first extract the rich information in the feature map and then gradually fuse the contextual feature information. These models often produce incomplete change maps due to gradual dilution of semantics during the progressive fusion. To enable effective information transfer between the feature maps of different layers of the network, we propose to replace the fusion mechanism in FPNs by aggregating the feature maps of different layers. Specifically, inspired by Li et al.^41^, we design the MSFA that adaptively predicts a set of weights based on the importance of different layer features. The purpose of this design is to effectively enhance the feature representation of the feature map.

**Figure 3:**
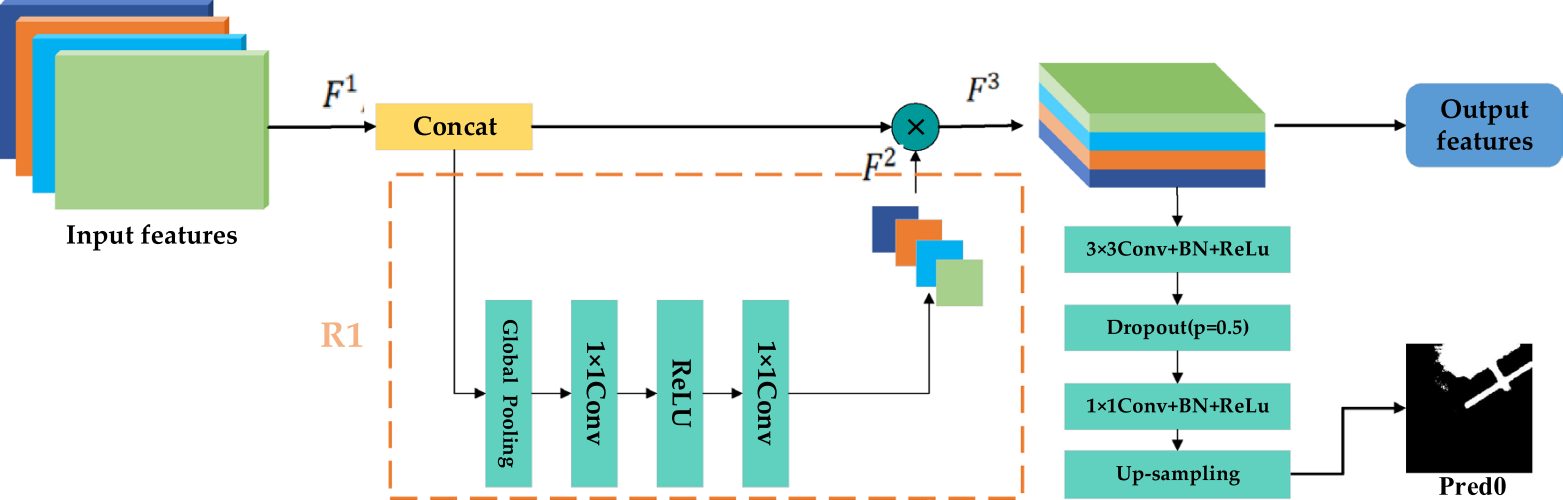
The structure of MSFA (created by ‘Microsoft Office Visio 2013’ url: https://www.microsoft.com/zh-cn/microsoft-365/previous-versions/microsoft-visio-2013).

The structure of MSFA is shown in Fig. 3. The size of the four feature maps outputted by LFFM is $X\in {\mathbb{R}}^{96\times H/4\times W/4}$, *H* and *W* denote the height and width of the original image. The four feature maps output from the LFFMs are concatenated along the channel dimension to obtain ${F}^{1}\in {\mathbb{R}}^{384\times H/4\times W/4}$. Subsequently, ${F}^{1}$ is compressed by global average pooling through the R1 branch, and then the adaptive weight coefficients ${F}^{2}\in {\mathbb{R}}^{384\times 1\times 1}$ at different levels are obtained by convolution and activation function, the calculation process is shown in the following equation:

6 $${F}^{2} ={f}^{1\times 1}[ReLU({f}^{1\times 1}\left(GAP\left({F}^{1}\right)\right))]$$

where the $GAP$ denotes the global average pooling, the $ReLU$ is the ReLU activation function, and the ${f}^{1\times 1}$ is the $1\times 1$ convolution layer. From the channel dimension, for different layers, a rich feature representation is obtained by adaptive aggregating the weights of different layers. Then, ${F}^{2}$ are multiplied with ${F}^{1}$ to obtain a feature map ${F}^{3}\in {\mathbb{R}}^{384\times H/4\times W/4}$ with different weight coefficients for each channel.

7 $${F}^{3}={F}^{2}\times C({F}^{1})$$

The ${F}^{1}$, ${F}^{2}$ and ${F}^{3}$ inside Eq. (7) are shown in Fig. 3. $C$ represents concatenation operation. With the MSFA, the high-level and low-level features can be fully fused, also providing sufficient object location information to the high-level features and rich semantic information to the low-level features. In this way, MSFA provides aggregated features that contain more semantic information and significant detail information, thus enabling the model to extract feature representations with rich information, which can solve the problem of inadequate feature extraction caused by the decreasing proportion of feature information carried by deep-level features in the overall fusion.

### Multi-scale feature distribution module

Although a rich feature representation as well as better detection results can be obtained after processing by the MSFA module, the prediction results are still unsatisfactory by using this single-stage inference, as shown in Fig. 9. Most of the existing fusion approaches for CD networks are based on a series of improvements of the FPN model. However, these methods tend to result in inaccurate localization of change regions or poor change region boundaries, since the high-level features captured by deeper layers may be gradually diluted, and the low-level features learned from shallow layers are insufficient to detect precise change regions throughout the progressive feature fusion.

In this study, we propose to combine the aggregated feature map with the progressive structure of FPN again. Since the MSFA module performs feature aggregation for different stages, the feature map after aggregation can contain feature representations of different stage information in the backbone network. Progressive fusion of FPNs on top of this will significantly alleviate the limitations of the fusion approach with FPNs alone. Therefore, we design an MSFD module to allocate multi-layer features by multi-scale pooling. This enables the semantic and positional information in the feature maps to be fully accessible at each level, which contributes to the feature fusion in FPN and facilitates the model to detect the exact region of change. The feature maps after the MSFA module combine multi-scale feature representation, and the MSFD in this paper assigns multi-level features by multi-scale pooling of aggregated features. The model structure of MSFD is shown in Fig. 1, from which it can be seen that the feature map F1 is concerned with the details of objects in the image, and the ability to identify the boundary of the change region can be enhanced by extracting the detail information in the feature map. The prediction map Pred1 output from the MSFD structure contains rich semantic information and edge detail information, which helps to better detect change regions. Meanwhile, as shown in Fig. 12 of the experimental part of this paper, the feature map can effectively detect the edge information of the change region after MSFD processing, which proves the effectiveness of MSFD and the ability of the model to detect the edge information of the change region.

Specifically, the MSFD module first feeds the feature map outputs from MSFA module to the average pooling layers with pyramid down-sampling rates to convert the aggregated features to different scale spaces. As shown in Fig. 1, the down-sampling rates are {8, 4, 2, 1, 1} from top to bottom, and then these five layers are respectively passed through $1\times 1$, $3\times 3$, and $5\times 5$ convolution operations to obtain five feature maps with different sizes and the same number of channels, which, from top to bottom, are sequentially fused by up-sampling features to obtain the prediction map Pred1. It should be noted that at the end of the progressive fusion, a pixel classifier consisting of a convolution layer and Dropout (p = 0.5) is used, and the structure of the main role is to change the number of channels of the features and, to some extent, serves to prevent overfitting. By this fusion, since the distributed feature maps at each fusion level simultaneously incorporate semantics and fine details, more discriminative and complementary representations can be well preserved along the progressive fusion path. The fusion effect is thus greatly enhanced for achieving superior performance.

### Loss function

In this paper, three CD public datasets, CDD, WHU-CD and LEVIR-CD, were used to evaluate the proposed method. This is because the proportion of changed and unchanged pixels varies greatly in these three datasets, and the changed pixels represent only a small fraction of the unchanged pixels. Considering issues such as pixel imbalance that can be biased in the training network, the network proposed in this paper uses the loss function (BCL) proposed by STANet^17^ to optimize the network parameters. The change map output by the network represents a batch of binary label maps, where 0 represents unchanged pixels and 1 represents changed pixels. With the MSFA module, we obtain a global change difference map, and with the MSFD, we obtain a local change difference map. The loss function of this model is shown as follows.

8 $$L=\beta {L}_{{D}_{1}^{*}}+\gamma {L}_{{D}_{2}^{*}}$$

In this equation, we calculate the loss of the global difference map Pred1 and the local difference map Pred0 of the network output with label respectively. ${L}_{{D}_{1}^{*}}$ and ${L}_{{D}_{2}^{*}}$ are summed to become the loss function for training this network, and the effect of different coefficient shares on the network is considered by setting the $\upbeta$ and $\upgamma$ parameters. The effect of setting different ratios of $\upbeta$ and $\upgamma$ on the model can be seen in the ablation experiment. The loss function ${L}_{{D}_{1}^{*}}$ and ${L}_{{D}_{2}^{*}}$ are similar, as shown in Eq. (9).

9 $$\begin{array}{c}L\left({D}^{*},{M}^{*}\right)=\lambda \times \frac{1}{{n}_{u}}\sum\limits_{b,i,j}\left(1-{M}_{b,i,j}^{*}\right){D}_{b,i,j}^{*}+\left(1-\lambda \right)\times \frac{1}{{n}_{c}}\sum\limits_{b,i,j}{M}_{b,i,j}^{*}Max\left(0,m-{D}_{b,i,j}^{*}\right)\end{array}$$

In Eq. (9), $b$, $i$ and $j$ and represent batch, height and width, and m is set to 2. Considering the ratio of unchanged pixels and changed pixels, we set $\lambda$ to 0.7 in the experiment. ${n}_{c}$ and ${n}_{u}$ are the numbers of changed and unchanged pixels. The calculation formula is as follows.

10 $$\begin{array}{c}{n}_{u}=\sum\limits_{b,i,j}1-{M}_{b,i,j}^{*}\end{array}$$

11 $$\begin{array}{c}{n}_{c}=\sum\limits_{b,i,j}{M}_{b,i,j}^{*}\end{array}$$

## Experiments and analysis

In the experiments, we evaluate the effectiveness of the proposed MFPF-Net using three publicly available datasets. We first introduce the three datasets used in this paper, followed by the evaluation metrics and detailed setup of the experiments. Finally, the experiment is analyzed in detail.

### Datasets

With the continuous development of remote sensing satellite technology, some high-quality remote sensing CD datasets have emerged in recent years. The publicly available remote sensing image CD datasets are useful for comparing the performance of different CD methods. We conduct experiments on three widely used CD benchmark datasets, including CDD^16^, LEVIR-CD^17^, and WHU-CD^35^.

**Figure 4:**
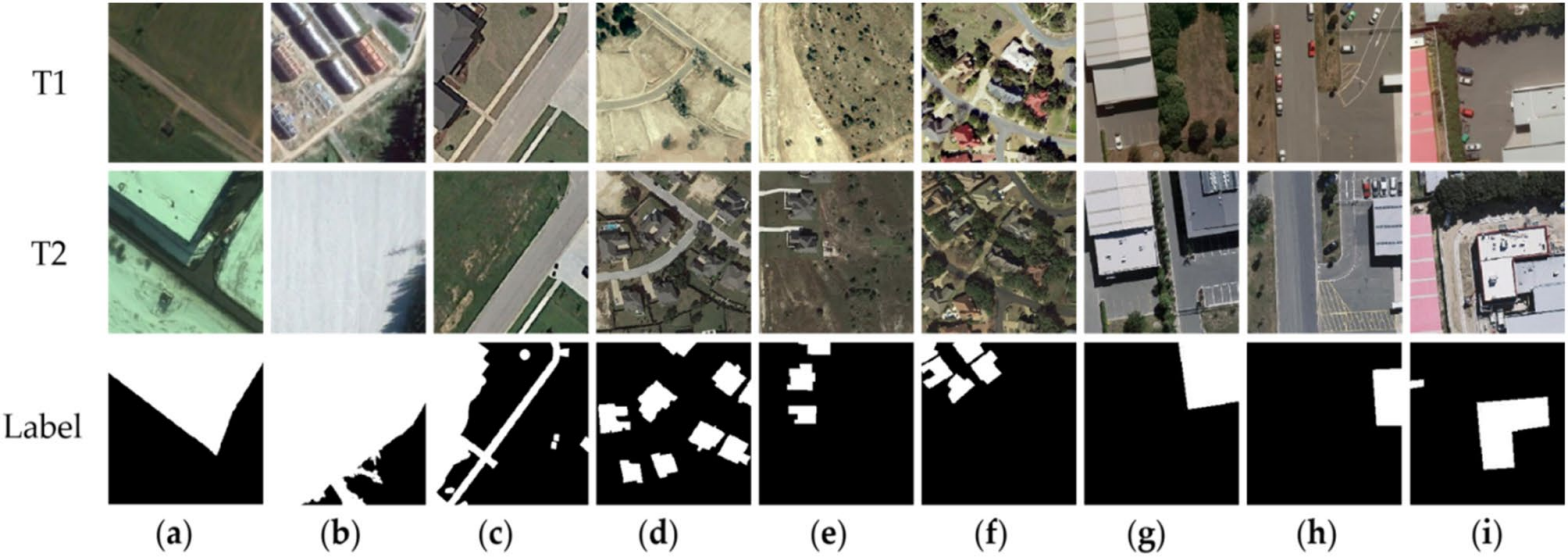
Bi-temporal remote sensing images from three open-source datasets CDD, WHU-CD and LEVIR-CD. The T1 are change before images, the T2 are change after images, the label represents the changed areas and the unchanged areas. (**a–c**) The images in the CDD dataset. (**d–f**) The images in the LEVIR-CD dataset. (**g–i**) The images in the WHU-CD dataset (created by ‘Microsoft Office Visio 2013’ url: https://www.microsoft.com/zh-cn/microsoft-365/previous-versions/microsoft-visio-2013).

The CDD dataset was acquired by Google Earth in 2018. It consists of seven pairs of $4725 \times 2200$ pixels seasonal variation images without appendages and four pairs of $1900 \times 1000$ pixels seasonal variation images with appendages. The authors of the paper divided the images into non-overlapping $256\times 256$ pixels image pairs, and the changing objects include cars, large building structures, etc. The dataset contains 16,000 pairs of seasonal images, of which 10,000 pairs are the training set, and 3,000 pairs are the validation set and test set, respectively. Figure 4 shows some of these samples.

The LEVIR-CD provided by researchers consists of 637 pairs of remote sensing satellite images of resolution 0.5 m per pixel. The size of the LEVIR-CD dataset is $1024\times 1024$, and the span of the dataset is from 2002 to 2018. These changes include several cities in Texas, such as Austin, Lakeway, Bee Cave, etc. For the limitation of GPU memory capacity, we cropped $1024\times 1024$ images to $256\times 256$. Consequently, we get 7120 pairs of the training set, 1024 pairs of the validation set, and 2048 pairs of the test set, respectively. Some examples of the LEVIR dataset are shown in Fig. 4.

The WHU-CD dataset is a public dataset for CD with $32507\times 15354$ pixels provided by^35^. The image resolution of the dataset is 0.075 and its time is from 2012 to 2016. Considering the limitation of memory usage, we cut the WHU-CD dataset into 9947 non-overlapping images of $224\times 224$ size. Finally, the dataset consists of 7957 pairs of the training set, 995 pairs of the validation set, and 995 pairs of the test set. Some examples of the WHU-CD dataset are shown in Fig. 4.

### Evaluation metrics and settings

In this paper, to compare the difference between the label maps and the predicted change maps, we use four evaluation metrics precision (P), recall (R), overall accuracy (OA), and F1-score (F1) to evaluate the efficiency of the proposed method. In the CD task, the higher P denotes the more accuracy of detected changed pixels and the higher R represents the greater ability of the model to find more changed pixels. OA denotes the overall accuracy. F1 is a metric for measuring the accuracy of the binary classification model. It considers the P and R of the classification model at the same time. The value of F1 ranges from 0 to 1, the higher the value, the better the performance of the model.

12 $$\begin{array}{c}P=\frac{TP}{TP+FP}\end{array}$$

13 $$\begin{array}{c}R=\frac{TP}{TP+FN}\end{array}$$

14 $$\begin{array}{c}{\text{F}}{1}=\frac{2}{{\text{P}}^{-1}\text{+}{\text{R}}^{-1}}\end{array}$$

15 $$\begin{array}{c}{\text{O}}{\text{A}}=\frac{\text{TP+TN}}{\text{TP+FP+TN+FN}}\end{array}$$

In the above equation, as shown in Table 1, TP is the number of correctly detected changed pixels, TN represents the number of correctly detected unchanged pixels, FP is the number of false alarm pixels, and FN is the number of lost unchanged pixels.

**Table 1:** The detailed explanation of TN, TP, FN, and FP.

True value	predicted value	
	Positive	Negative
Positive	TP	FN
Negative	FP	TN

In our experiment, we used the Ubuntu 16.04 ($\times 86$) operating system and a single NVIDIA Tesla V100 graphics processing unit. The proposed method is implemented by Pytorch with python3.6 as the backend. Adam (${\upbeta }_{1}=0.5$, ${\upbeta }_{2}=0.999$) is selected to optimize network parameters, and the entire training period is set to 200 epochs. The initial learning rate is 0.001 in the first 100 epochs, in the next 100 epochs, the value of the learning rate decays linearly to 0.

### Comparison of experimental results

**Figure 5:**
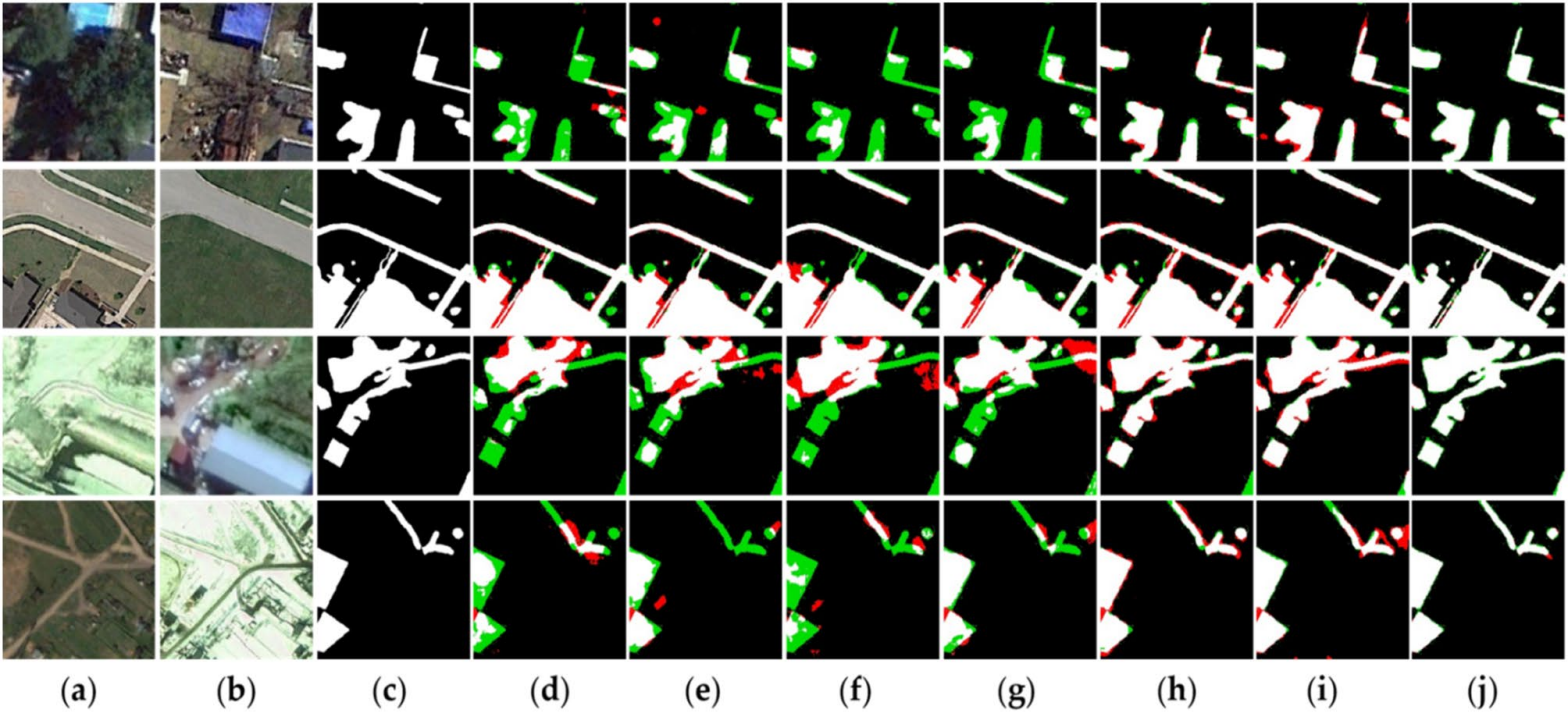
Bi-temporal remote sensing images from CDD dataset. The (**a**) are change before images; the (**b**) are change after images; the (**c**) represents the changed areas and the unchanged areas; (**d**) are results obtained by CDNet; (**e**) are results obtained by FC-EF; (**f**) are results obtained by FC-Siam-Diff; (**g**) are results obtained by FC-Siam-Conc; (**h**) are results obtained by DASNet; (**i**) are results obtained by STANet; (**j**) are results obtained by MFPF-Net (created by ‘Microsoft Office Visio 2013’ url: https://www.microsoft.com/zh-cn/microsoft-365/previous-versions/microsoft-visio-2013).

**Figure 6:**
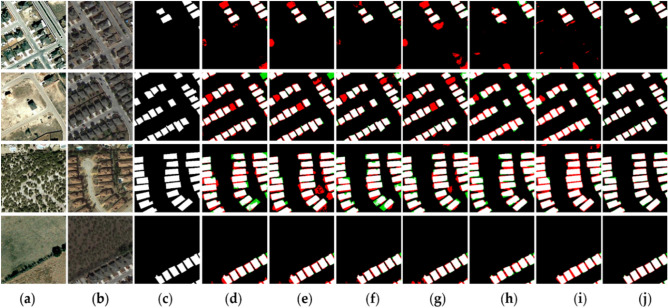
Bitemporal remote sensing images from LEIVR-CD dataset. The (**a**) are change before images; the (**b**) are change after images; the (**c**) represents the changed areas and the unchanged areas; (**d**) are results obtained by CDNet; (**e**) are results obtained by FC-EF; (**f**) are results obtained by FC-Siam-Diff; (**g**) are results obtained by FC-Siam-Conc; (**h**) are results obtained by DASNet; (**i**) are results obtained by STANet; (**j**) are results obtained by MFPF-Net (created by ‘Microsoft Office Visio 2013’ url: https://www.microsoft.com/zh-cn/microsoft-365/previous-versions/microsoft-visio-2013).

**Figure 7:**
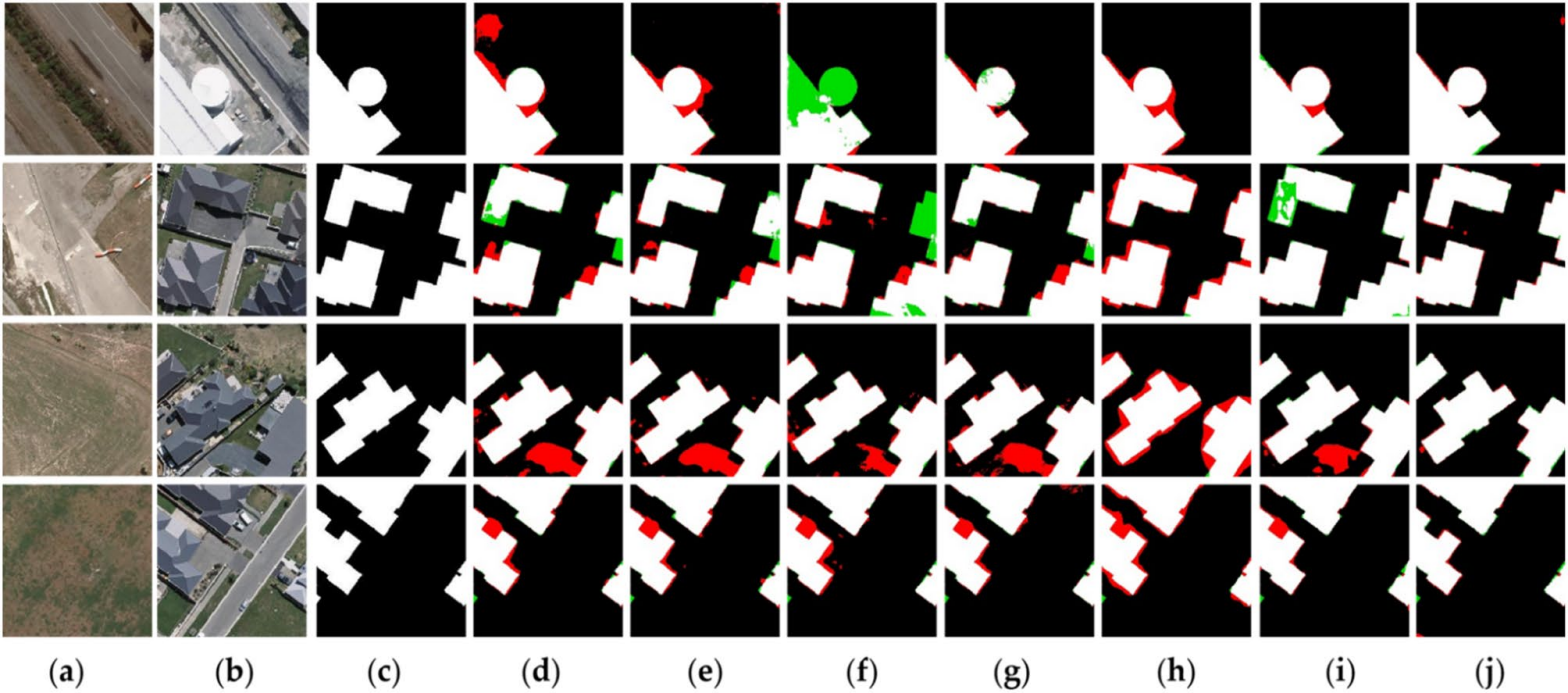
Bi-temporal remote sensing images from WHU-CD dataset. The (**a**) are change before images; the (**b**) are change after images; the (**c**) represents the changed areas and the unchanged areas; (**d**) are results obtained by CDNet; (**e**) are results obtained by FC-EF; (**f**) are results obtained by FC-Siam-Diff; (**g**) are results obtained by FC-Siam-Conc; (**h**) are results obtained by DASNet; (**i**) are results obtained by STANet; (**j**) are results obtained by MFPF-Net (created by ‘Microsoft Office Visio 2013’ url: https://www.microsoft.com/zh-cn/microsoft-365/previous-versions/microsoft-visio-2013).

In the experiment, this study compares the proposed method with six classical CD methods, which include CDNet^37^, FC-EF^38^, FC-Siam-Conc^38^, FC-Siam-Diff^38^, DASNet^10^, and STANet^17^. The comparative experiments of the six models on three datasets are shown in Figs. 5, 6 and 7. In the scenes shown in these three datasets, black and white pixels represent unchanged and changed areas, respectively. Simultaneously, the red pixels are false detections, and the green pixels are missed detections. Specific examples can be given based on the images. The results show that both large scenes and small objects can be well detected in the changing regions of the bi-temporal images, and the shape and boundary clarity of the changing regions are highly consistent with the ground truth. This paper analyzes the CD effect of this method from qualitative and quantitative aspects.

The drawing tools used in this paper are Microsoft Visio 2013 and MATLAB R2019a. They are available at the following links: https://www.microsoft.com/zh-cn/microsoft-365/previous-versions/microsoft-visio-2013 and https://www.mathworks.cn/products/matlab.html, respectively.

#### CDD dataset

We selected several representative scenes from the CDD dataset that are affected by factors such as season and lighting and compared them with six other SOTA CD models.

The performance of different models is shown in Fig. 5. The white indicates true positive, the black indicates true negative, the red indicates false positive, and the green indicates false negative. It is clear from the analysis that the proposed method is very sensitive to object changes and can capture small changes that are not detected in other methods. Compared with other methods, the proposed method significantly reduces the number of incorrectly detected and missed pixels. As can be seen from Fig. 5, there are lots of green missed detection regions in the CDNet, FC-EF, FC-Siam-Conc, and FC-Siam-Diff visualizations, as well as some red false detection regions. In CDNet, the detection performance of the model is poor due to the deficiency of model design in feature layer fusion. The attention mechanism is used in both DASNet and STANet. In the model, the attention mechanism is used to extract rich feature information, and the detection effect is significantly improved compared with the models of CDNet, FC-EF, FC-Siam-Conc, and FC-Siam-Diff (sample Fig. 5d–g). However, the boundary information of the change region in these two models has not been accurately detected. As shown in Fig. 5, there are also many red areas in the picture, indicating that models have false detections.

As can be seen from Fig. 5, these four images contain some changes in buildings and roads. When there is a small area of change, MFPF-Net can almost identify all small areas of change. There are also some noises in the image at T1 that affect the detection performance. The proposed network can also filter these noises, and the returned change map matches the label. Compared with other models, the proposed method can overcome the influence of irrelevant factors such as seasonal changes and illumination on the model to the maximum extent, and accurately detects the edge detail information of changing objects compared with the label.

Next, as shown in Table 2, we evaluate the model performance from the quantitative results of the evaluation indicators. Specifically, on the CDD data set, the R, P, F1, and OA of the method are 96.4%, 95.3%, 95.9%, and 99.0% respectively. Compared with the excellent remote sensing CD methods STANet and DASNet, the F1 of the method reaches 95.9%, increased by 4.4% and 3.4% respectively. The P of the MFPF-Net reaches 95.3%, which is 8.3% and 3.3% higher than STANet and DASNet, respectively. Although the R of the MFPF-Net is similar to the STANet, we have achieved significant improvements in the other three indicators. From the data point of view, the performance of the MFPF-Net is much better than that of FC-EF, FC-Siam-Conc, and FC-Siam-Diff models.

**Table 2:** Comparison of CDD dataset results (the best performance is emphasized in bold).

Method	R (%)	P (%)	F1(%)	OA(%)
CDNet	81.7	82.7	82.2	96.4
FC-EF	76.1	81.5	77.1	94.1
FC-Siam-Diff	83.6	85.8	83.7	95.8
FC-Siam-Conc	82.5	84.4	82.5	95.7
DASNet	93.0	92.0	92.5	98.1
STANet	**96.5**	87.0	91.5	97.9
MFPF-Net (ours)	96.4	**95.3**	**95.9**	**99.0**

#### LEIVR-CD dataset

We selected several representative scenarios from the LEVIR-CD dataset and compared them with six other advanced CD models. The performance of different models is shown in Fig. 6. The proposed method has made a breakthrough in capturing small changes that are not detected in other methods. Compared with other methods, the proposed method significantly reduces the number of false detections and missed pixels. As can be seen from the visualization of comparison models (sample Fig. 6d–i) there are a large number of red false detection regions and also a small amount of green missed detection regions in Fig. 6.

It can be seen from Fig. 6 that the four images are all about the changes of buildings and the change maps are relatively regular. Due to the influence of weather, the image quality at the T2 moment is relatively poor, but MFPF-Net has performed well under this condition, and all the change areas in the label map are detected, but there is little edge information not detected. Compared with other models, the proposed method considers adequate fusion of the deep and shallow effective information of the feature, so MFPF-Net basically accurately detects the edge details of the changed areas, although there are a small number of missing and false detection areas.

As shown in Table 3, on the LEVIR-CD dataset, the proposed method has 89.6%, 89.9%, 89.8%, and 99.0% in the four metrics of R, P, F1, and OA, respectively. Compared with the STANet with good performance, the MFPF-Net improves 7.3%, 3.7%, and 0.5% in the three metrics of P, F1, and OA, respectively, although the value is slightly lower in R. Compared with DASNet, MFPF-Net is 1.7%, 8.4%, 4.2%, and 1.6% higher in R, P, F1, and OA, respectively. At the same time, it can be seen from Table 3 that the performance of the proposed model is significantly higher than that of CDNet, FC-EF, FC-Siam-Diff, and FC-Siam-Conc. Taken together, the proposed method has a significant improvement in all aspects and experiments with superior performance.

**Table 3:** Comparison of LEVIR-CD dataset results (the best performance is emphasized in bold).

Method	R (%)	P (%)	F1(%)	OA(%)
CDNet	89.1	74.6	81.2	97.1
FC-EF	85.6	76.5	80.8	97.9
FC-Siam-Diff	87.5	79.8	83.5	98.2
FC-Siam-Conc	83.9	81.6	82.7	98.2
DASNet	87.9	81.5	84.6	98.4
STANet	**89.9**	82.6	86.1	98.5
MFPF-Net (Ours)	89.6	**89.9**	**89.8**	**99.0**

#### WHU-CD dataset

We selected a few representative scenarios from the WHU-CD dataset and compared them with six other advanced CD models. The performance of different models is shown in Fig. 7. The proposed method can capture small changes that are not detected in other methods. Compared with other methods, the proposed method significantly reduces the number of false detections and missed pixels. In Fig. 7f, it can be seen that there are more green missed detection regions in the FC-EF visualization maps, and also some red false detection regions. There are more false detection regions (Fig. 7d–h). In STANet, the spatial–temporal attention mechanism is used to strengthen feature extraction, which improves the performance of the model to some extent. Therefore, the visualization maps in STANet are significantly improved, but there are also false detections in terms of inaccurate detection of boundary information in the change region.

It can be seen from Fig. 7 that the four images are all about the changes of buildings, and the shapes of the label are different, especially the circular changes. MFPF-Net has a very good detection effect on all the change regions, especially the irregular shape, and there are many missing and false detections in other models. The proposed network can completely detect the edge information of the change regions. Compared with other models, the MFPF-Net accurately detects the edge detail information of the changing building objects in Fig. 7 and obtains a good performance.

As shown in Table 4, on the WHU-CD dataset, the proposed method achieves 92.1%, 93.2%, 92.7%, and 99.3% in the four metrics of R, P, F1, and OA, respectively. Compared with the well-performing DASNet algorithm, the MFPF-Net improves 1.2%, 2.8%, 2%, and 0.3% in the four metrics of R, P, F1, and OA, respectively. Compared with the other five widely used algorithms in the table, the proposed method achieves SOTA results.

**Table 4:** Comparison of WHU-CD dataset results (the best performance is emphasized in bold).

Method	R (%)	P (%)	F1(%)	OA(%)
CDNet	84.2	75.6	79.7	97.9
FC-EF	74.6	84.1	79.1	98.1
FC-Siam-Diff	86.8	84.2	85.5	98.6
FC-Siam-Conc	84.5	84.3	84.4	98.5
DASNet	90.9	90.4	90.7	99.0
STANet	86.3	92.6	89.4	99.0
MFPF-Net (ours)	**92.1**	**93.2**	**92.7**	**99.3**

### Ablation study

To demonstrate the effectiveness of our proposed method, we performed on three datasets CDD, LEVIR-CD, and WHU-CD a series of ablation experiments.

#### Effectiveness of three innovation modules.

**Figure 8:**
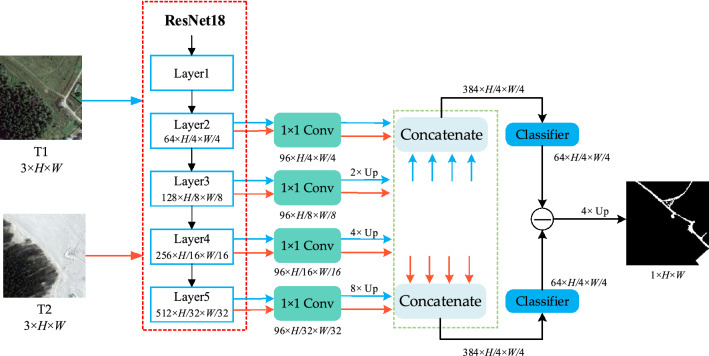
The structure of baseline (created by ‘Microsoft Office Visio 2013’ url: https://www.microsoft.com/zh-cn/microsoft-365/previous-versions/microsoft-visio-2013).

First, we gradually added each proposed module to the Baseline and finally integrated all the modules, including LFFM, MSFA, and MSFD, together. The detailed structure of the Baseline is shown in Fig. 8. We conducted four ablation experiments on three datasets. There are four experiments: Baseline, Baseline + LFFM, Baseline + LFFM + MSFA, and Baseline + LFFM + MSFA + MSFD. Table 5 shows the results of these four experiments. It can be seen that without adding the three proposed innovative modules, the network performs poorly, with F1 of 89.8%, 82.9%, and 85.3% on the three datasets CDD, LEVIR-CD, and WHU-CD, respectively, which is a huge gap compared to other models that join the innovative modules.

**Table 5:** Ablation experiment of innovative modules.

Settings				CDD				LEVIR				WHU-CD			
Baseline	LFFM	MSFA	MSFD	R	P	F1	OA	R	P	F1	OA	R	P	F1	OA
√				95.0	85.2	89.8	97.5	89.5	77.1	82.9	98.1	92.8	78.9	85.3	98.4
√	√			**96.8**	91.8	94.2	98.6	**95.3**	81.8	88.0	98.7	92.4	88.3	90.3	99.0
√	√	√		93.9	**95.8**	94.9	98.8	94.2	83.9	88.7	98.8	**93.2**	89.6	91.3	99.1
√	√	√	√	96.4	95.3	**95.9**	**99.0**	89.6	**89.9**	**89.8**	**99.0**	92.1	**93.2**	**92.7**	**99.3**

All the scores are described in percentage (%) (the best performance is emphasized in bold).

In the Baseline + LFFM model, the LFFM module fully fuses the bi-temporal features, which enables the model to obtain rich feature information. As can be seen from the data in the Table 5, all the indicators of the model have been significantly improved. In the CDD dataset, R, P, F1, and OA are improved by 1.8%, 6.6%, 4.4%, and 1.1%, respectively. In the LEVIR-CD dataset, R, P, F1, and OA improved by 5.8%, 4.6%, 5.1%, and 0.6%, respectively. In the WHU-CD dataset, R, F1, and OA improved by 9.4%, 5%, and 0.6%, respectively. It can be seen from the data that the proposed LFFM module has a significant effect on the detection accuracy improvement and facilitates adequate feature extraction.

In the Baseline + LFFM + MSFA model, we applied the MSFA module on top of the Baseline + LFFM, which can solve the problems caused by the FPN-like feature fusion mechanism to a certain extent due to the analysis above, and can also extract the key region features and obtain an enhanced feature representation, making the network robust. It can be seen from the three datasets that, compared with Baseline + LFFM, although the model has a decrease in R in the CDD and LEVIR-CD datasets, it has improved in three metrics: P, F1, and OA. the F1 metric, which is an important indicator of the comprehensive measure of remote sensing CD performance, has been improved in all three datasets and also can prove the effectiveness of our proposed MSFA module.

The addition of the MSFD to Baseline + LFFM + MSFA is the proposed network. With the addition of the MSFD module, the semantic information and location information in the feature maps are fully accessible at each level, facilitating the model to detect the precise change regions. Due to the characteristics of P–R curves, in general, the detection rate tends to be low when the accuracy is high and the detection rate tends to be low when the accuracy is high. Compared with the Baseline + LFFM and Baseline + LFFM + MSFA models, the P metric of the MFPF-Net network achieves good results, although the R metric decreases slightly. F1 and OA metrics achieve good performance, which proves that the MSFD module proposed by MFPF-Net can be used in combination with other modules to make further improvements in network performance.

**Figure 9:**
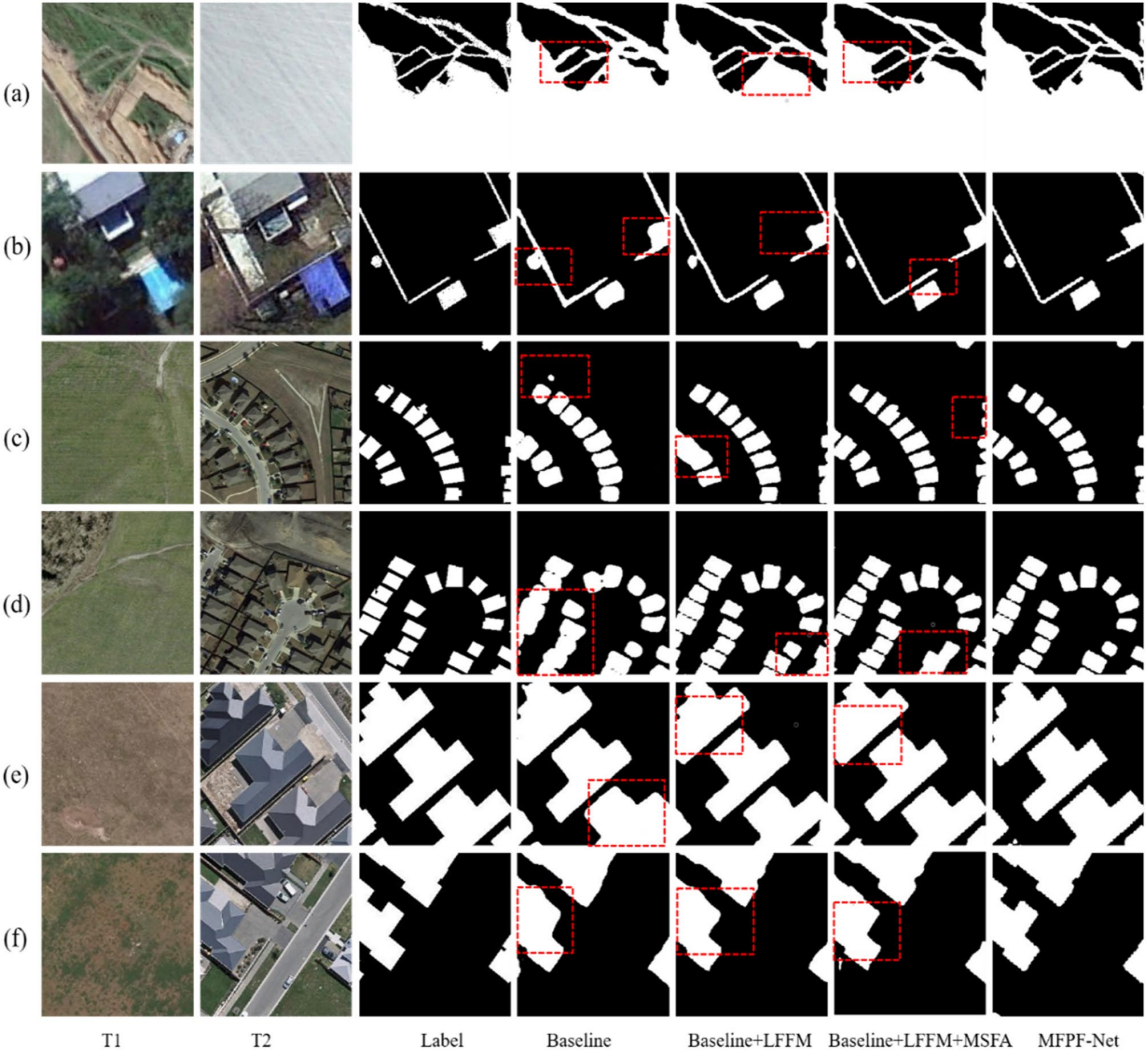
The ablation experimental images from three datasets. They should be listed as: (**a,b**) from CDD dataset; (**c,d**) from LEVIR-CD dataset; (**e,f**) WHU-CD dataset (created by ‘Microsoft Office Visio 2013’ url: https://www.microsoft.com/zh-cn/microsoft-365/previous-versions/microsoft-visio-2013).

Figure 9 shows the ablation experimental results. The red dotted lines in the figure are the parts with large differences between the change maps and Labels obtained by different models. It can be seen from Fig. 9 that the change maps generated by the MFPF-Net algorithm are more consistent with Labels in the three datasets. There are many missing and false detections in the models of Baseline, Baseline + LFFM, and Baseline + LFFM + MSFA, especially in the edge details of the changed areas.

#### Effect of the different proportional parameters of the loss function

**Figure 10:**
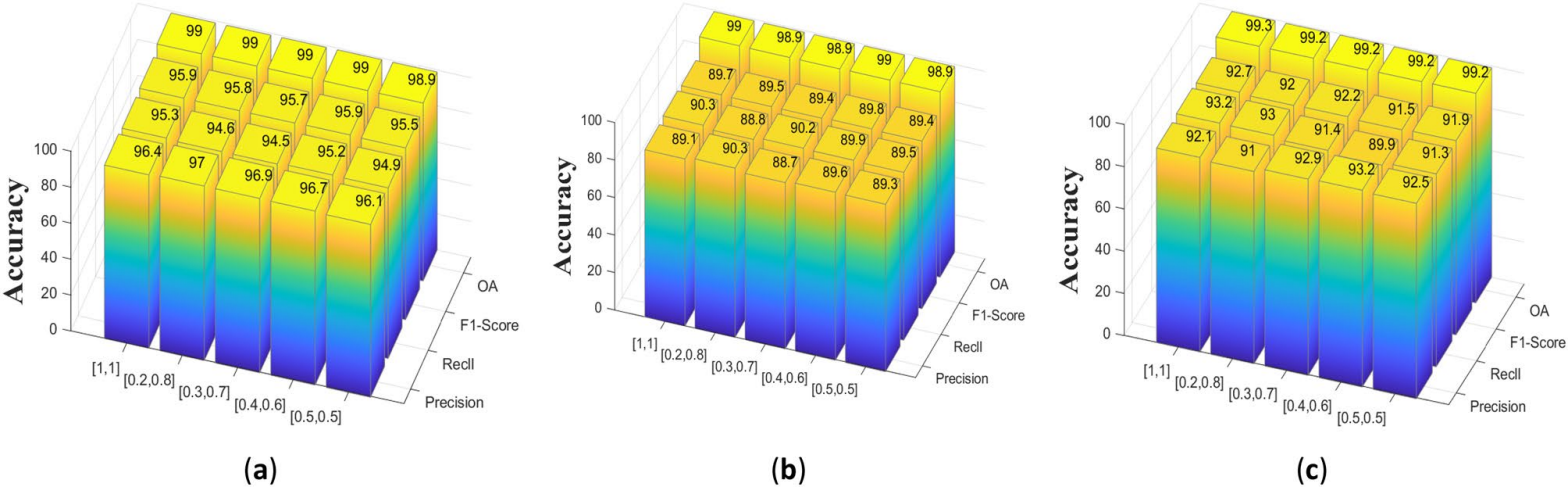
Setting different loss function proportional ablation experiments on three datasets, they should be listed as: (**a**) CDD dataset; (**b**) LEVIR-CD dataset; (**c**) WHU-CD dataset (created by “matlab R2019a” url: https://ww2.mathworks.cn/products/matlab.html).

In this experiment, we adopted a deeply supervised strategy for the loss function calculation from two prediction maps output by two modules, MSFA and MSFD, and to verify the effect of different percentages of deep supervision loss functions on the network model, we used different scale factors on the CDD, LEVIR-CD and WHU-CD datasets for validation, and the results are shown in Table 6 below. From Fig. 10, you can clearly and intuitively see the changes in the four indicators brought about by setting different proportions of the two loss functions. Collectively when $\upbeta =$ 1, $\upgamma =1$, the MFPF-Net achieves optimal performance in CDD and WHU-CD datasets, when $\upbeta =0.6$, $\upgamma =0.4$, the proposed method achieves optimal results LEVIR-CD dataset, but the performance of the proposed model is similar to that of $\upbeta =$ 1, $\upgamma =1.$ Based on the comprehensive performance of the three datasets, we take $\upbeta =$ 1, $\upgamma =1$.

**Table 6:** Different proportion results of loss function (the best performance is emphasized in bold).

Proportion		CDD				LEVIR-CD				WHU-CD			
$\Gamma$	$\upbeta$	R (%)	P (%)	F1 (%)	OA (%)	R (%)	P (%)	F1 (%)	OA (%)	R (%)	P (%)	F1 (%)	OA (%)
1	1	96.4	**95.3**	**95.9**	**99.0**	89.1	**90.3**	89.7	**99.0**	92.1	**93.2**	**92.7**	**99.3**
0.2	0.8	**97.0**	94.6	95.8	**99.0**	**90.3**	88.8	89.5	98.9	91.0	93.0	92.0	99.2
0.3	0.7	96.9	94.5	95.7	**99.0**	88.7	90.2	89.4	98.9	92.9	91.4	92.2	99.2
0.4	0.6	96.7	95.2	**95.9**	**99.0**	89.6	89.9	**89.8**	**99.0**	**93.2**	89.9	91.5	99.2
0.5	0.5	96.1	94.9	95.5	98.9	89.3	89.5	89.4	98.9	92.5	91.3	91.9	99.2

#### Impact of difference map

As mentioned before, most CD methods obtain the change map by concatenating two bi-temporal images. In this paper, based on a series of experimental data, we propose to concatenate bi-temporal feature pairs with their difference map and then use the convolution operation, this design is implemented in LFFM. For bi-temporal feature pairs, concatenating their difference map contributes to increasing the differences and avoiding gradient vanishing. This is because the difference map carries obvious difference information, change region feature information, and the weight of the unchanged region is effectively reduced, which helps to highlight the feature information of the changed region. At the same time, fully effective feature fusion of bi-temporal remote sensing images can be obtained in this way.

To demonstrate the effectiveness of this mechanism, we propose a comparison experiment. In experiment, the bi-temporal feature pairs are directly concatenated in series, and then the next operations are kept consistent. The experimental results are shown in Table 7.

**Table 7:** Impact of difference map (the best performance is emphasized in bold).

Difference map	CDD				LEVIR-CD				WHU-CD			
	R (%)	P (%)	F1 (%)	OA (%)	R (%)	P (%)	F1 (%)	OA (%)	R (%)	P (%)	F1 (%)	OA (%)
No difference map	**96.8**	93.8	95.3	98.9	86.0	**93.2**	89.4	**99.0**	**92.9**	90.0	91.5	**99.2**
Difference map	96.4	**95.3**	**95.9**	**99.0**	**89.6**	89.9	**89.8**	**99.0**	92.1	**93.2**	**92.7**	**99.3**

As can be seen in Table 7, in the model, using the difference map to concatenate with Image1 and Image2 could get better results than not using the difference map. It is easy to see that in most of the evaluation metrics, the model with difference map has higher scores than the model without difference map. In particular, the F1 score of the former is 0.6%, 0.4%, and 1.2% higher than that of the latter in the three datasets of CDD, LEVIR-CD, and WHU-CD respectively. Therefore, difference map is very important for the training of MFPF-Net.

#### Effect of the proposed MSFA

We analyzed the effectiveness of the designed MSFA module through several series of experiments on three datasets, and we made four sets of comparisons, and the first three sets were replaced by three other methods for the MSFA module in this network, which we named M1, M2, and M3. M4 represents our proposed MSFA module.

M1: The multi-scale fused feature maps output from LLFM are directly concatenated. Instead of generating a set of weights for the aggregated features, which means that part R1 in Fig. 3 is not used, the next operations are the same as the steps in Fig. 1.

M2: The multi-scale feature maps output by LFFM are directly concatenated, and then the global average pooling operation is performed on the aggregated feature map to obtain the weights and multiply them with the aggregated feature map. The subsequent operations are the same as those of the MFPF-Net network.

M3: We apply the R1 part in Fig. 3 to the features of each layer outputted from LFFM, then multiply them with the original features of each layer, and finally concatenate these feature maps. This is done independently at each layer before the concatenation. The subsequent operation is the same as that of the MFPF-Net network.

M4: We use our proposed MSFA module to learn the adaptive weights of each layer of stages, and subsequently perform aggregation of the information extracted from different stages.

**Figure 11:**
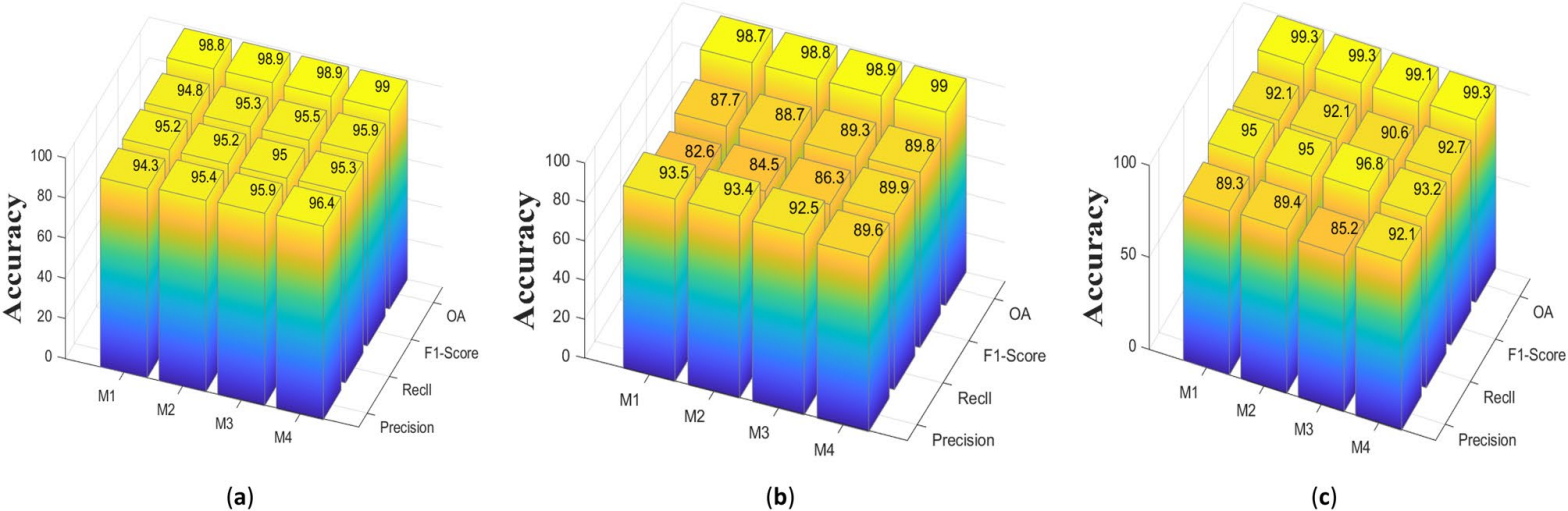
Experiments on three datasets show that MSFA achieves better performance than other settings, they should be listed as: (**a**) CDD dataset; (**b**) LEVIR-CD dataset; (**c**) WHU-CD dataset (created by “matlab R2019a” url: https://ww2.mathworks.cn/products/matlab.html).

Table 8 reflects the results obtained from the above four different operations in the three datasets. It can be observed that M2 and M3 methods are generally better than the M1 method, which shows that the R1 branch we used in the MSFA module is effective in improving the CD performance. It is found that the performance of M2 and M3 is inferior to that of M4 because the design of M2 does not consider the importance of information between different stages, and M3 ignores the dependency between the original feature map and the weight-allocated feature map. From the data of M4, it can be obtained that our proposed MSFA also achieves significant performance in improving the CD accuracy. At the same time, we can see from Fig. 11 that our proposed LFFM module has superior performance, using the M4 module overall performance is better than using M1, M2, and M3 modules.

**Table 8:** Ablation experiment of MSFA module (the best performance is emphasized in bold).

Module	CDD				LEVIR-CD				WHU-CD			
	R (%)	P (%)	F1 (%)	OA (%)	R (%)	P (%)	F1 (%)	OA (%)	R (%)	P (%)	F1 (%)	OA (%)
M1	94.3	95.2	94.8	98.8	**93.5**	82.6	87.7	98.7	89.3	95.0	92.1	**99.3**
M2	95.4	95.2	95.3	98.9	93.4	84.5	88.7	98.8	89.4	95.0	92.1	99.3
M3	95.9	95.0	95.5	98.9	92.5	86.3	89.3	98.9	85.2	**96.8**	90.6	99.1
M4	**96.4**	**95.3**	**95.9**	**99.0**	89.6	**89.9**	**89.8**	**99.0**	**92.1**	93.2	**92.7**	**99.3**

#### Effect of the proposed MSFD

**Figure 12:**
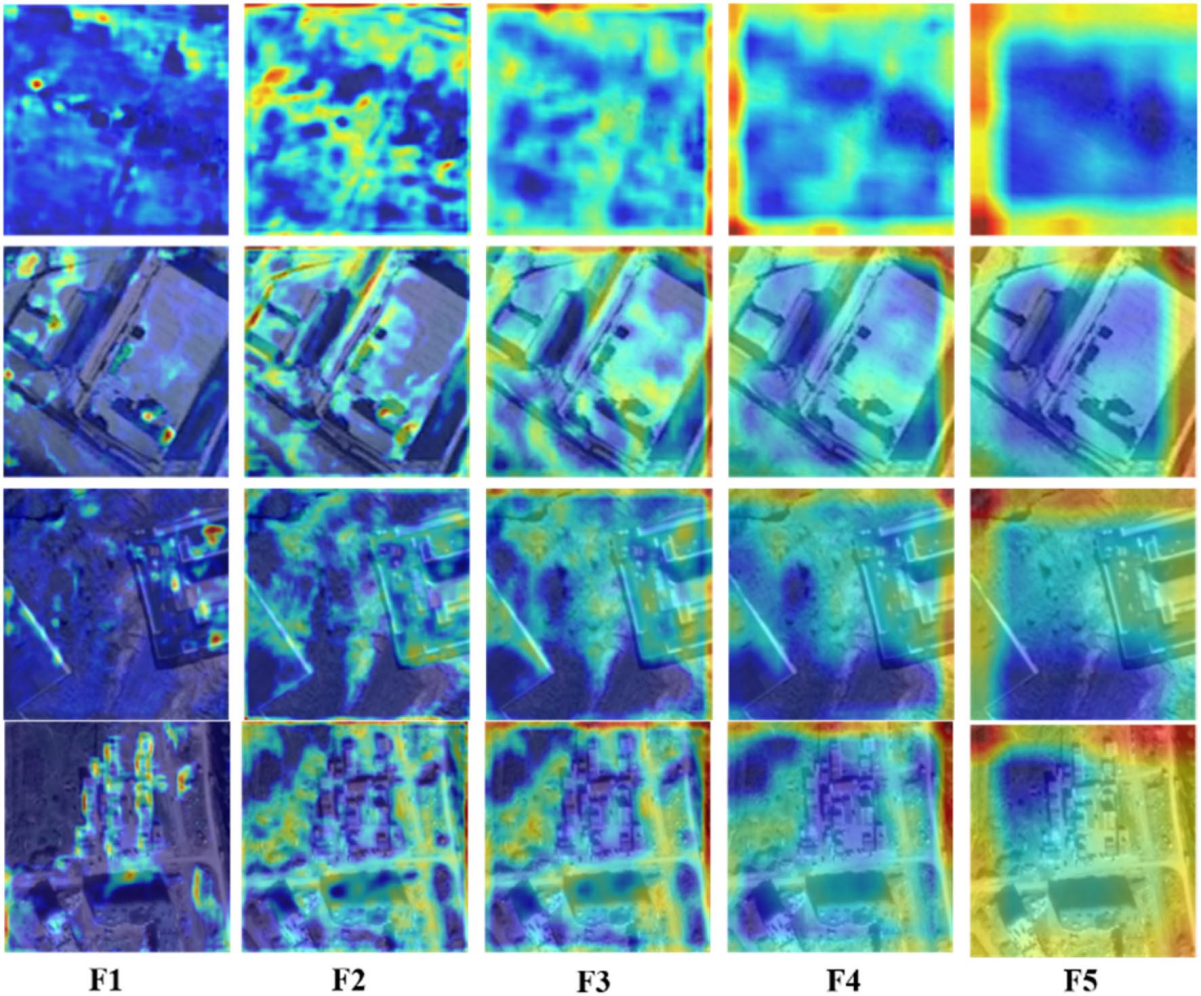
Visualization of feature maps output from different scale pooling layers on CDD dataset (created by ‘Microsoft Office Visio 2013’ url: https://www.microsoft.com/zh-cn/microsoft-365/previous-versions/microsoft-visio-2013).

To further explore the role of the MSFD module in the MFPF-Net, this paper uses the Grad-CAM^43^ tool to analyze. The Grad-CAM tool will mark areas that the model considers important. The redder the marked area, the more the model focuses on the area, which can effectively evaluate whether the model fully extracts the features of the changed areas. Figure 12 shows the visualized feature maps of the feature maps after passing through the MSFD layers, and F1-F5 shows the heat map of the output feature maps after the MSFD module branches at the markers in Fig. 1. The feature maps after the MSFA module combine the multi-scale feature representation, and the MSFD in this paper allocates the multi-level features by performing multi-scale pooling on the aggregation features. It can be seen from the images that the feature map F1 focuses on the details of the object in the image, F5 focuses on the edge contour information in the image, and the range of attention from F1 to F5 is expanding. Semantic information and edge details can be accessed adaptively at each fusion level in the MSFD module, which helps to better detect change areas.

### Accuracy/efficiency trade-offs

We first evaluate the performance of the model on the CDD dataset according to the time complexity and spatial complexity. In this article, Table 9 lists the time consumption and model parameters of all methods. T/E represents time/epoch, T/P represents time/parameters. In Table 9 "time/parameters" represents the efficiency of the model. The lower the value, the better the trade-off between time complexity and space complexity. In addition, F1 and OA are selected to reflect the accuracy of the model, which can better explain the comprehensive performance of the model.

**Table 9:** Performance and speed trade-offs (the best performance is emphasized in bold).

Method	Train					Test
	F1 (%)	OA (%)	T/E	Parameter	T/P ($\times {10}^{2}$ s/M)	Test time (3000 images)
CDNet	82.2	96.4	~ 1879s	~ **1.28 M**	14.68	~ 1020 s
FC-EF	77.1	94.1	~ 978 s	~ 1.47 M	6.65	** ~ 253 s**
FC-Siam-Diff	83.7	95.8	~ 1134 s	~ 1.51 M	7.51	~ 287 s
FC-Siam-Conc	82.5	95.7	~ 1207 s	~ 1.62 M	7.45	~ 288 s
STANet	89.9	97.6	~ 564 s	~ 16.93 M	0.33	~ 576 s
DASNet	91.5	97.9	1380 s	~ 39.60 M	0.35	~ 407 s
MFPF-Net	**95.9**	**99.0**	** ~ 324 s**	~ 39.78 M	**0.08**	~ 296 s

As shown in Table 9, CDNet has the fewest model parameters, but the efficiency of the model is poor and its accuracy is also average. FC-Siam-Diff and FC-Siam-Conc achieve similar accuracy, but they are inefficient and time-consuming. FC-EF model has a small number of parameters and the least time consumption of test pictures, but it does not perform well in performance. The parameter quantity and time consumption of STANet are increased compared with those of FC series models, but the F1 and OA of STANet have been greatly improved. Compared with STANet, DASNet model has further improved F1 and OA, but it also brings the problem of increasing the number of parameters. In addition, the parameters of MFPF-Net are large, but it only needs about 296 s to generate the change map of the whole test set, which is equivalent to only about 0.098 s to obtain every 256 × 256 change maps, which is acceptable for most CD tasks. At the same time, the proposed MFPF-Net model also achieves good performance on F1 and OA. In conclusion, the efficiency of MFPF-Net is competitive with several SOTA methods.

## Conclusions

In this paper, a novel deep learning network for remote sensing image CD is proposed, named MFPF-Net. To fully fuse the feature maps of each layer of bi-temporal images by layer, a layer feature fusion module (LFFM) is designed. LFFM emphasizes the fusion of same-layer bi-temporal feature maps and their difference maps, which focuses on the change regions information while retaining some detailed information. We discuss the problems caused by the FPN-like feature fusion mechanism, based on which the MSFA feature fusion mechanism is proposed. This mechanism can perform feature aggregation for different stages while generating adaptive parameters to highlight feature information in changing regions. Finally, multi-level pooling operation is performed in the MSFD module and combined with FPN, where the feature maps of each layer have the semantics of the information of other layers, which makes the progressive fusion of multi-scale feature maps more effective. The efficient combination of our proposed three models reduces the information loss during feature extraction and enables the network to achieve SOTA performance. In this study, we analyze some problems in extracting features and then propose a deeply supervised CD network for high-resolution remote sensing images. The proposed network is improved for the problem of feature extraction. The network achieves superior results on three datasets, CDD, LEVIR-CD, and WHU-CD, and also proves the effectiveness and feasibility of the MFPF-Net.

Although MFPF-Net solves to some extent the problems of missed and false detection prevalent in remote sensing image CD, the number of model parameters is large and the MFPF-Net network is based on a deeply supervised strategy, which requires abundant model training time. Further exploration and research on model light-weighting and unsupervised can be carried out in future work.

## Data Availability

The CDD, LEVIR-CD, WHU-CD datasets are openly available at: https://drive.google.com/fifile/d/1GX656JqqOyBi_Ef0w65kDGVto-nHrNs9 (accessed on 8 April 2022), https://justchenhao.github.io/LEVIR/ (accessed on 8 April 2022), http://gpcv.whu.edu.cn/data/building_dataset.html (accessed on 8 April 2022), respectively.
